# Supplementary material for: DoReMiTra: an R/Bioconductor data package for orchestrating the analysis of radiation transcriptomic studies
Source: Bioinform Adv. 2026 Mar 28;6(1):vbag092. doi: 10.1093/bioadv/vbag092 (PMC13143432; doi:10.1093/bioadv/vbag092)
Supplement: vbag092_Supplementary_Data [file vbag092_supplementary_data.pdf]

Using DoReMiTra for analyzing and integrating publicly available transcriptomics radiation datasets

Supplementary file for 'DoReMiTra: An R/Bioconductor data package for orchestrating the analysis of radiation transcriptomic studies'

AUTHORS
Ahmed Salah
Sebastian Zahnreich
Federico Marini

AFFILIATIONS
Ahmed Salah: Department of Radiation Oncology and Radiation Therapy, Mainz, Germany; Institute of Medical Biostatistics, Epidemiology and Informatics (IMBEI), Mainz, Germany
Sebastian Zahnreich: Department of Radiation Oncology and Radiation Therapy, Mainz, Germany
Federico Marini: Institute of Medical Biostatistics, Epidemiology and Informatics (IMBEI), Mainz, Germany

PUBLISHED
March 7, 2026

1 Loading the required packages

Here we load all packages required to reproduce the analysis in this notebook.

```
library("DoReMiTra")
library("mosdef")
library("org.Hs.eg.db")
library("DESeq2")
library("ggplot2")
library("ComplexHeatmap")
library("VennDiagram")
library("limma")
library("circlize")
library("GeneTonic")
library("clusterProfiler")
library("patchwork")
```

2 Listing all available datasets

Users can use the `list_DoReMiTra_datasets()` function to have a concise overview of all datasets currently available in the DoReMiTra collection. It returns a structured data.frame including dataset identifiers and key descriptive information, allowing users to quickly inspect the available resources before retrieval.

```
datasets <- list_DoReMiTra_datasets()
knitr::kable(datasets)
```

Dataset	RadiationType	Organism	ExpSetting	Accession	Tissue	Autl
SE_Amundson_2019_InVivo_GSE124612_GPL11202	gamma ray	Mus musculus	InVivo	GSE124612	Blood	Amu
SE_Aryankalayil_2018_InVivo_GSE104121_GPL10787	gamma ray	Mus musculus	InVivo	GSE104121	Blood	Arya
SE_Aryankalayil_2018_InVivo_GSE104121_GPL21163	gamma ray	Mus musculus	InVivo	GSE104121	Blood	Arya
SE_Park_2017_ExVivo_GSE102971_GPL10332_MacacaMulatta	gamma ray	Macaca mulatta	ExVivo	GSE102971	Blood	Park
SE_Park_2017_ExVivo_GSE102971_GPL10332_HomoSapiens	gamma ray	Homo sapiens	ExVivo	GSE102971	Blood	Park
SE_Vasilyev_2017_ExVivo_GSE97000_GPL17077	gamma ray	Homo sapiens	ExVivo	GSE97000	Blood	Vasi
SE_Paul_2013_ExVivo_GSE44201_GPL6480	gamma ray	Homo sapiens	ExVivo	GSE44201	Blood	Paul
SE_Paul_2013_ExVivo_GSE44201_GPL6848	gamma ray	Homo sapiens	ExVivo	GSE44201	Blood	Paul
SE_Nosel_2013_ExVivo_GSE43151_GPL13497	gamma ray	Homo sapiens	ExVivo	GSE43151	Blood	Nos
SE_Girardi_2012_ExVivo_GSE20173_GPL6480	gamma ray	Homo sapiens	ExVivo	GSE20173	Blood	Gira

Dataset	RadiationType	Organism	ExpSetting	Accession	Tissue	Aut
SE_Amundson_2011_ExVivo_GSE23515_GPL6480	gamma ray	Homo sapiens	ExVivo	GSE23515	Blood	Amu
SE_Gruel_2008_ExVivo_GSE6978_GPL4803	gamma ray	Homo sapiens	ExVivo	GSE6978	Blood	Gru
SE_Rouchka_2019_ExVivo_GSE63952_GPL15207	gamma ray	Homo sapiens	ExVivo	GSE63952	Blood	Rou
SE_Amundson_2008_ExVivo_GSE8917_GPL1708	gamma ray	Homo sapiens	ExVivo	GSE8917	Blood	Amu
SE_Manikandan_2014_ExVivo_GSE36355_GPL6883	gamma ray	Homo sapiens	ExVivo	GSE36355	Blood	Mar
SE_Lee_2013_ExVivo_GSE44245_GPL570	gamma ray	Homo sapiens	ExVivo	GSE44245	Blood	Lee
SE_Rouchka_2015_ExVivo_GSE64375_GPL6244	gamma ray	Homo sapiens	ExVivo	GSE64375	Blood	Rou
SE_Ankermit_2015_ExVivo_GSE55953_GPL14550	gamma ray	Homo sapiens	ExVivo	GSE55953	Blood	Ank
SE_Broustas_2018_InVivo_GSE113509_GPL11202	Neutron; X-ray	Mus musculus	InVivo	GSE113509	Blood	Brou
SE_Broustas_2017_InVivo_GSE85323_GPL10333	Neutron; X-ray	Mus musculus	InVivo	GSE85323	Blood	Brou
SE_Broustas_2017_ExVivo_GSE90909_GPL13497	Neutron; X-ray	Homo sapiens	ExVivo	GSE90909	Blood	Brou
SE_Paul_2010_InVivo_GSE23393_GPL6480	X-ray	Homo sapiens	InVivo	GSE23393	Blood	Paul
SE_Ghandhi_2015_ExVivo_GSE65292_GPL13497	X-ray	Homo sapiens	ExVivo	GSE65292	Blood	Gha
SE_Flores_2009_ExVivo_GSE15341_GPL8332	X-ray	Homo sapiens	ExVivo	GSE15341	Blood	Flor
SE_Amundson_2011_InVivo_GSE20162_GPL6480	X-ray	Homo sapiens	InVivo	GSE20162	Blood	Amu
SE_Broustas_2023_InVivo_GSE196400_GPL11202	X-ray	Mus musculus	InVivo	GSE196400	Blood	Brou
SE_Broustas_2022_InVivo_GSE184361_GPL11202	X-ray	Mus musculus	InVivo	GSE184361	Blood	Brou
SE_Broustas_2021_InVivo_GSE132559_GPL11202	X-ray	Mus musculus	InVivo	GSE132559	Blood	Brou
SE_Yamaguchi_2020_InVivo_GSE137192_GPL1261	X-ray	Mus musculus	InVivo	GSE137192	Blood	Yam
SE_Mukherjee_2019_InVivo_GSE114142_GPL11202	X-ray	Mus musculus	InVivo	GSE114142	Blood	Muk
SE_Amundson_2018_InVivo_GSE101402_GPL11202	X-ray	Mus musculus	InVivo	GSE101402	Blood	Amu
SE_Amundson_2018_InVivo_GSE99176_GPL11202	X-ray	Mus musculus	InVivo	GSE99176	Blood	Amu
SE_Paul_2015_InVivo_GSE62623_GPL10333	X-ray	Mus musculus	InVivo	GSE62623	Blood	Paul
SE_Ghandhi_2018_InVivo_GSE84898_GPL13497	X-ray	Macaca mulatta	InVivo	GSE84898	Blood	Gha
SE_Broustas_2021_InVivo_GSE133451_GPL11202	X-ray	Mus musculus	InVivo	GSE133451	Blood	Brou
SE_Salah_2025_ExVivo	X-ray	Homo sapiens	ExVivo	NA	Blood	Sala

3 Listing metadata information

The `list_DoReMiTra_metadata_fields()` function provides an overview of the available sample-level metadata fields across all datasets included in DoReMiTra. For each metadata variable, the function reports the unique values observed across the compendium.

This utility facilitates intuitive dataset exploration and helps users identify appropriate filtering criteria before retrieving datasets with search_DoReMiTra_datasets() or get_DoReMiTra_dataset(). In particular, it allows users to quickly inspect supported categories such as radiation type, dose, time point, organism, tissue, and other study-specific annotations.

```
list_DoReMiTra_metadata_fields()
```

```
#> Available metadata fields:
#> - RadiationType: gamma ray, Neutron; X-ray, X-ray
#> - Organism: Homo sapiens, Macaca mulatta, Mus musculus
#> - ExpSetting: ExVivo, InVivo
#> - Accession: GSE101402, GSE102971, GSE104121, GSE113509, GSE114142, GSE124612, GSE132559, (
#> - Tissue: Blood
#> - Author: Amundson, Ankermit, Aryankalayil, Broustas, Flores, Ghandhi, Girardi, Gruel, Lee,
```

4 Searching for datasets

The user can use `search_DoReMiTra_datasets()` to filter the available DoReMiTra datasets based on metadata fields such as radiation type, organism, author, or experimental setting. This function helps narrow down datasets of interest before fetching them.

```
# X-ray datasets
search_DoReMiTra_datasets(radiation_type = "X-ray",
                          organism = "Homo sapiens",
                          exp_setting = "ExVivo")
#> Filtering for radiation type: X-ray
#> Filtering for organism: Homo sapiens
#> Filtering for experimental setting: ExVivo
#> Author not provided - searching across all authors.
#>
#> Matching datasets found: 4
#> To retrieve one or more of these datasets, you can use:
#> se_name1 <- get_DoReMiTra_data("SE_Broustas_2017_ExVivo_GSE90909_GPL13497")
#> se_name2 <- get_DoReMiTra_data("SE_Ghandhi_2015_ExVivo_GSE65292_GPL13497")
#>
#> For more details, please refer to `?get_DoReMiTra_data`
#> [1] "SE_Broustas_2017_ExVivo_GSE90909_GPL13497"
#> [2] "SE_Ghandhi_2015_ExVivo_GSE65292_GPL13497"
#> [3] "SE_Flores_2009_ExVivo_GSE15341_GPL8332"
#> [4] "SE_Salah_2025_ExVivo"

# gamma ray datasets
search_DoReMiTra_datasets(radiation_type = "gamma ray",
                          organism = "Homo sapiens",
                          exp_setting = "ExVivo")
#> Filtering for radiation type: gamma ray
#> Filtering for organism: Homo sapiens
#> Filtering for experimental setting: ExVivo
#> Author not provided - searching across all authors.
#>
#> Matching datasets found: 14
#> To retrieve one or more of these datasets, you can use:
#> se_name1 <- get_DoReMiTra_data("SE_Park_2017_ExVivo_GSE102971_GPL10332_HomoSapiens")
#> se_name2 <- get_DoReMiTra_data("SE_Vasilyev_2017_ExVivo_GSE97000_GPL17077")
#>
#> For more details, please refer to `?get_DoReMiTra_data`
#>  [1] "SE_Park_2017_ExVivo_GSE102971_GPL10332_HomoSapiens"
#>  [2] "SE_Vasilyev_2017_ExVivo_GSE97000_GPL17077"
#>  [3] "SE_Paul_2013_ExVivo_GSE44201_GPL6480"
#>  [4] "SE_Paul_2013_ExVivo_GSE44201_GPL6848"
#>  [5] "SE_Nosel_2013_ExVivo_GSE43151_GPL13497"
#>  [6] "SE_Girardi_2012_ExVivo_GSE20173_GPL6480"
#>  [7] "SE_Amundson_2011_ExVivo_GSE23515_GPL6480"
#>  [8] "SE_Gruel_2008_ExVivo_GSE6978_GPL4803"
#>  [9] "SE_Rouchka_2019_ExVivo_GSE63952_GPL15207"
#> [10] "SE_Amundson_2008_ExVivo_GSE8917_GPL1708"
#> [11] "SE_Manikandan_2014_ExVivo_GSE36355_GPL6883"
#> [12] "SE_Lee_2013_ExVivo_GSE44245_GPL570"
#> [13] "SE_Rouchka_2015_ExVivo_GSE64375_GPL6244"
#> [14] "SE_Ankermit_2015_ExVivo_GSE55953_GPL14550"
```

5 Retrieving the datasets

After selecting the dataset of interest, `get_DoReMiTra_data()` can be used to fetch a `SummarizedExperiment` (SE) object with stable gene identifiers and consistently formatted sample-level metadata directly from ExperimentHub.

```
# we start with a recent study by Salah et al., conducted on RNA-seq
dataset_name_1 <- "SE_Salah_2025_ExVivo"

# we would like also to compare it to another dataset from another radiation type to see share
dataset_name_2 <- "SE_Paul_2013_ExVivo_GSE44201_GPL6848"
```

```
se_salah <- get_DoReMiTra_data(dataset_name_1)
#> see ?DoReMiTra and browseVignettes('DoReMiTra') for documentation
#> loading from cache
se_paul <- get_DoReMiTra_data(dataset_name_2, gene_symbol = TRUE)
#> see ?DoReMiTra and browseVignettes('DoReMiTra') for documentation
#> loading from cache
```

These simple commands provide the user an analysis-ready object for the datasets generated by (Salah et al. 2025) and by (Paul, Smilenov, and Amundson 2013).

6 Obtaining summaries of the datasets

To quickly obtain a summary of an SE object, use `summarize_DoReMiTra_se()`, which provides a concise overview including the number of samples, genes, radiation types, tissue, etc.

```
summarize_DoReMiTra_se(se_salah)
#> This dataset is generated by: Salah
#>     Platform: RNAseq
#>     Organism(s): Homo sapiens
#>     Radiation Type: X-ray
#>     Experiment Setting: ExVivo
#>     Number of Samples: 30
#>     Tissue: Blood
#>     Accession: NA
#>     For more information about this study, please check:
#> https://github.com/AhmedSAHassan/Phybion_ShortReads_Analysis
summarize_DoReMiTra_se(se_paul)
#> This dataset is generated by: Paul
#>     Platform: Microarray
#>     Organism(s): Homo sapiens
#>     Radiation Type: gamma ray
#>     Experiment Setting: ExVivo
#>     Number of Samples: 50
#>     Tissue: Blood
#>     Accession: GSE44201
#>     For more information about this study, please check:
#> https://www.ncbi.nlm.nih.gov/geo/query/acc.cgi?acc=GSE44201
```

Once we retrieved the analysis-ready datasets of intereset, we can explore them and analyse them in detail.

7 Comparing the metadata of multiple datasets

To compare key metadata information from two or more SE objects from the DoReMiTra collection, `compare_DoReMiTra_datasets()` summarizes key metadata information such as radiation type, dose, and time point.

```
se_list<- list(
  `Salah et al.` = se_salah,
  `Paul et al.` = se_paul
)
compare_DoReMiTra_datasets(se_list = se_list) |>
  kableExtra::kable()
```

	Salah et al.	Paul et al.
Radiation_type	X-ray	gamma ray
Dose	0.5Gy, 0Gy, 1Gy, 2Gy, 4Gy	0.5Gy, 0Gy, 2Gy, 5Gy, 8Gy
Sex	Female, Male	female, male
Time_point	2hrs, 6hrs	24hr, 6hr
Organism	Homo sapiens	Homo sapiens
Tissue	Blood	Blood
Sample_number	30	50

8 Using DoReMiTraExplorer for exploratory data analyses and visualization

One can also explore the datasets with `DoReMiTraExplorer`, a companion app package we have developed.

The command to install the app is exemplified in the following chunk:

```
# Install the DoReMiTra-explorer Shiny App
devtools::install_github("AhmedSAHassan/DoReMiTraExplorer")
```

The retrieved SummarizedExperiment objects can be explored with `DoReMiTra_explorer()`, an interactive Shiny application for visualization and exploratory analysis of DoReMiTra datasets. This app allows users to examine transcriptomic responses to radiation using:

- Principal Component Analysis (PCA)
- Boxplots for the distribution of expression values
- Dose-response gene plots
- Heatmaps of highly variable genes

It provides flexibility for both high-level overviews and detailed inspection of radiation-induced transcriptional patterns.

To load the app, we can simply call

```
# load the DoReMiTraExplorer package
library("DoReMiTraExplorer")

# launch the app if in an interactive session
if(interactive()) {
  DoReMiTraExplorer::DoReMiTra_explorer(se = se_salah)
}
```

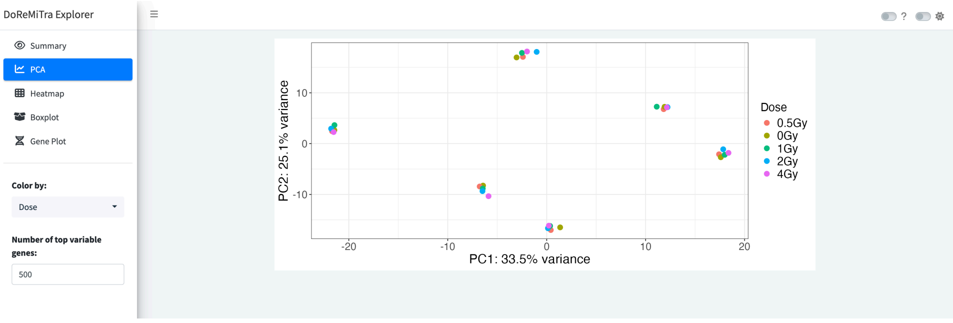

Snapshot of the DoReMiTraExplorer app for PCA plot

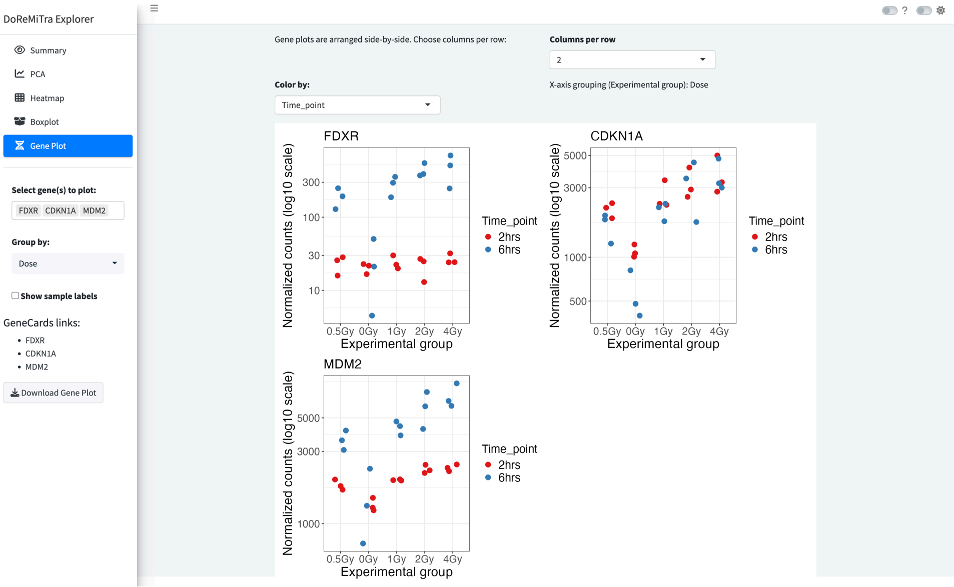

Snapshot of the DoReMiTraExplorer app dose-response gene plot

9 Analyzing the datasets, in detail

Once we identified and retrieved the analysis-ready datasets of intereset, we can explore and analyse them.

9.1 Working on the X-ray dataset

As we could observe from the PCA plot obtained via `DoReMiTraExplorer`, the donor and time effects are the main sources of variability. These factors should be accounted for in downstream statistical analyses. Specifically, we use in this case the DESeq2 statistical framework, and focus on the **2Gy vs 0Gy** comparison, starting from the subset of (Salah et al. 2025) including samples 2 hours after irradiation.

This dataset consists of 3 donors, 5 doses (0, 0.5, 1, 2, and 4Gy), and 2 timepoints (2h and 6h)

```
dds_salah <- DESeqDataSet(se_salah, design = ~1)
#> using counts and average transcript lengths from tximeta

dds_salah$Dose <- factor(dds_salah$Dose)
dds_salah$Donors <- factor(dds_salah$Donors)
dds_salah$Dose <- relevel(dds_salah$Dose, ref = "0Gy")

dds_salah_2h <- dds_salah[, dds_salah$Time_point == "2hrs"]
dds_salah_6h <- dds_salah[, dds_salah$Time_point == "6hrs"]

vsd_salah <- vst(dds_salah)
#> using 'avgTxLength' from assays(dds), correcting for library size

pca_plot <- plotPCA(vsd_salah,
                    intgroup = c("Donors", "Time_point"),
                    returnData = TRUE)
#> using ntop=500 top features by variance

percentVar <- round(100 * attr(pca_plot, "percentVar"))

PCA <- ggplot(pca_plot, aes(x = PC1, y = PC2, color = Donors, shape = Time_point)) +
  geom_point(size = 3) +
  scale_shape_manual(values = c(16, 17)) +
  xlab(paste0("PC1: ", percentVar[1], "% variance")) +
  ylab(paste0("PC2: ", percentVar[2], "% variance")) +
  theme_minimal()+
  theme(plot.background = element_rect(fill = "white", color = NA))

PCA
```

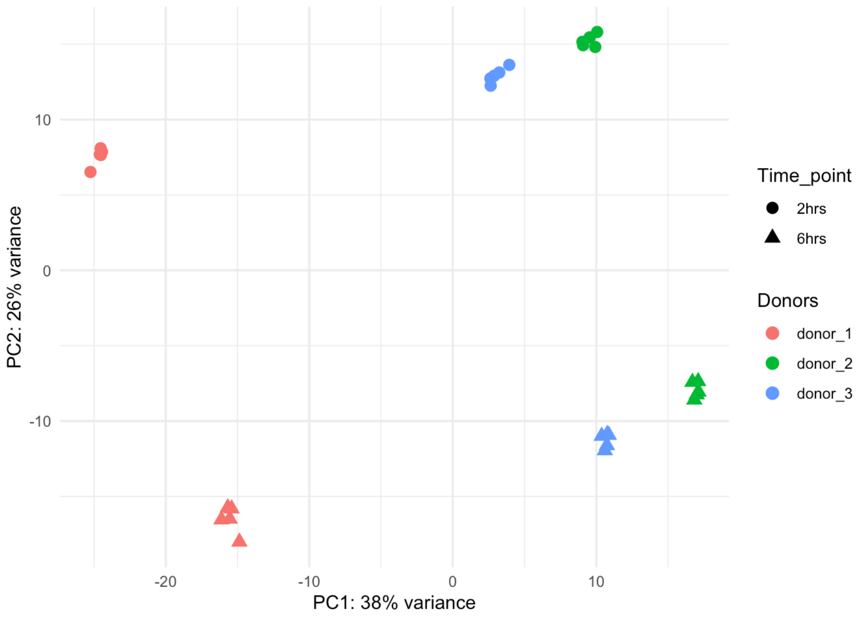


```
vsd_salah_6h <- vst(dds_salah_6h)
#> using 'avgTxLength' from assays(dds), correcting for library size

pca_donors <- plotPCA(vsd_salah_6h,
        intgroup = "Donors",
        returnData = FALSE) +
  theme_minimal() +
  labs(title = "Salah et al., PCA by donors")
#> using ntop=500 top features by variance
pca_dose <- plotPCA(vsd_salah_6h,
        intgroup = "Dose",
        returnData = FALSE) +
  theme_minimal() +
  labs(title = "Salah et al., PCA by dose")
#> using ntop=500 top features by variance

pca_donors | pca_dose
```

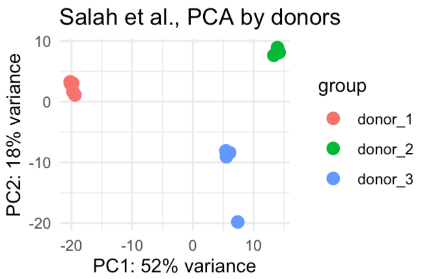

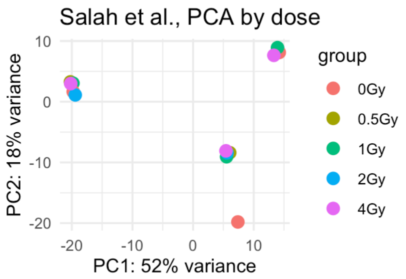

We quickly inspect a heatmap of the highly variable genes - as we have strong donor effect, we will account for it (removing that via `removeBatchEffect()`) for better visualization. The uncorrected raw counts will be used for the subsequent steps.

```
vsd_corrected <- vsd_salah_6h
assay(vsd_corrected) <- limma::removeBatchEffect(assay(vsd_corrected), batch = vsd_corrected$D
#> design matrix of interest not specified. Assuming a one-group experiment.
doses <- as.numeric(sub("Gy.*", "", colnames(vsd_corrected)))
col_order <- order(doses)
vsd_corrected <- vsd_corrected[, col_order]
colnames(vsd_corrected)
#>  [1] "0Gy-6hrs-do1"   "0Gy-6hrs-do2"   "0Gy-6hrs-do3"   "0.5Gy-6hrs-do1"
#>  [5] "0.5Gy-6hrs-do2" "0.5Gy-6hrs-do3" "1Gy-6hrs-do1"   "1Gy-6hrs-do2"
#>  [9] "1Gy-6hrs-do3"   "2Gy-6hrs-do1"   "2Gy-6hrs-do2"   "2Gy-6hrs-do3"
#> [13] "4Gy-6hrs-do1"   "4Gy-6hrs-do2"   "4Gy-6hrs-do3"

top_n <- 50
rv <- rowVars(assay(vsd_corrected))
top_genes <- order(rv, decreasing = TRUE)[1:top_n]
mat <- assay(vsd_corrected)[top_genes, ]
mat_scaled <- t(scale(t(mat)))
annotation <- as.data.frame(colData(vsd_corrected)[, c("Sex","Dose"), drop = FALSE])
Heatmap(mat_scaled,
        name = "expression",
        cluster_columns = FALSE,
        top_annotation = HeatmapAnnotation(df = annotation))
```

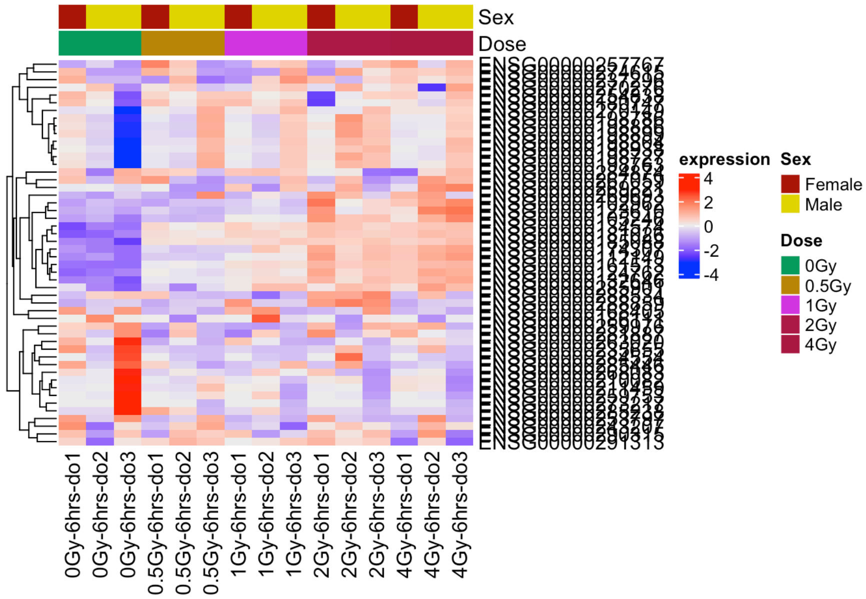

The columns of the heatmap are left ordered by dose.

Afterwards, we proceed with the modeling and testing step with DESeq2.

```r
dds_salah_6h
#> class: DESeqDataSet
#> dim: 62646 15
#> metadata(9): tximetaInfo quantInfo ... DoReMiTra version
#> assays(3): counts abundance avgTxLength
#> rownames(62646): ENSG00000000003 ENSG00000000005 ... ENSG00000293599
#>   ENSG00000293600
#> rowData names(3): gene_id tx_ids SYMBOL
#> colnames(15): 0.5Gy-6hrs-do1 1Gy-6hrs-do1 ... 2Gy-6hrs-do3 4Gy-6hrs-do3
#> colData names(11): Dose Time_point ... Platform Tissue

design(dds_salah_6h)<- ~Donors + Dose
dds_salah_6h <- DESeq(dds_salah_6h)
#> estimating size factors
#> using 'avgTxLength' from assays(dds), correcting for library size
#> estimating dispersions
#> gene-wise dispersion estimates
#> mean-dispersion relationship
#> final dispersion estimates
#> fitting model and testing
resultsNames(dds_salah_6h)
#> [1] "Intercept"                 "Donors_donor_2_vs_donor_1"
#> [3] "Donors_donor_3_vs_donor_1" "Dose_0.5Gy_vs_0Gy"
#> [5] "Dose_1Gy_vs_0Gy"           "Dose_2Gy_vs_0Gy"
#> [7] "Dose_4Gy_vs_0Gy"

# we will focus on one contrast here, 2Gy vs 0Gy
results_salah<- results(dds_salah_6h,
                        name = "Dose_2Gy_vs_0Gy",
                        alpha = 0.05)
summary(results_salah)
#>
#> out of 30201 with nonzero total read count
#> adjusted p-value < 0.05
#> LFC > 0 (up)       : 197, 0.65%
#> LFC < 0 (down)     : 77, 0.25%
#> outliers [1]       : 0, 0%
#> low counts [2]     : 14915, 49%
#> (mean count < 7)
#> [1] see 'cooksCutoff' argument of ?results
#> [2] see 'independentFiltering' argument of ?results
results_salah_shrink <- lfcShrink(dds_salah_6h,
                                  res = results_salah,
                                  coef = "Dose_2Gy_vs_0Gy")
#> using 'apeglm' for LFC shrinkage. If used in published research, please cite:
#>     Zhu, A., Ibrahim, J.G., Love, M.I. (2018) Heavy-tailed prior distributions for
#>     sequence count data: removing the noise and preserving large differences.
#>     Bioinformatics. https://doi.org/10.1093/bioinformatics/bty895

result_salah_sig <- as.data.frame(results_salah_shrink)
result_salah_sig <-
  result_salah_sig[!is.na(results_salah_shrink$padj) & result_salah_sig$padj <= 0.05, ]

# adding SYMBOL column
result_salah_sig$SYMBOL <- mapIds(
  org.Hs.eg.db,
  keys = rownames(result_salah_sig),
  column = "SYMBOL",
  keytype = "ENSEMBL",
  multiVals = "first"
)
#> 'select()' returned 1:many mapping between keys and columns

result_salah_sig |>
  head(10) |>
  knitr::kable()
```

	baseMean	log2FoldChange	lfcSE	pvalue	padj	SYMBOL
ENSG00000009413	1466.8788	0.8194507	0.2331190	0.0000219	0.0032261	REV3L
ENSG00000010818	3634.3748	0.6828260	0.1626106	0.0000016	0.0003454	HIVEP2
ENSG00000012779	2322.2113	0.3859968	0.1307390	0.0003284	0.0236759	ALOX5
ENSG00000013810	1421.9630	-0.5529292	0.1858397	0.0001786	0.0156867	TACC3
ENSG00000023445	2664.3304	1.0234267	0.2760269	0.0000082	0.0014388	BIRC3
ENSG00000023892	1837.3742	-0.5147620	0.1777516	0.0002644	0.0205193	DEF6
ENSG00000026103	802.8594	1.2610716	0.2076139	0.0000000	0.0000000	FAS
ENSG00000033178	781.7486	0.8676891	0.2883771	0.0001036	0.0101479	UBA6
ENSG00000035664	499.3658	-1.0199059	0.2264002	0.0000003	0.0000846	DAPK2
ENSG00000054148	223.0434	2.3754658	0.1837554	0.0000000	0.0000000	PHPT1

We can also visualize the up and down regulated genes via a volcano plot

```
mosdef::de_volcano(
  res_de = results_salah_shrink,
  mapping = "org.Hs.eg.db",
  labeled_genes = 50,
  logfc_cutoff = 0
)
#> 'select()' returned 1:many mapping between keys and columns
#> Warning: Removed 32445 rows containing missing values or values outside the scale range
#> (`geom_point()`).
#> Warning: Removed 62597 rows containing missing values or values outside the scale range
#> (`geom_text_repel()`).
```

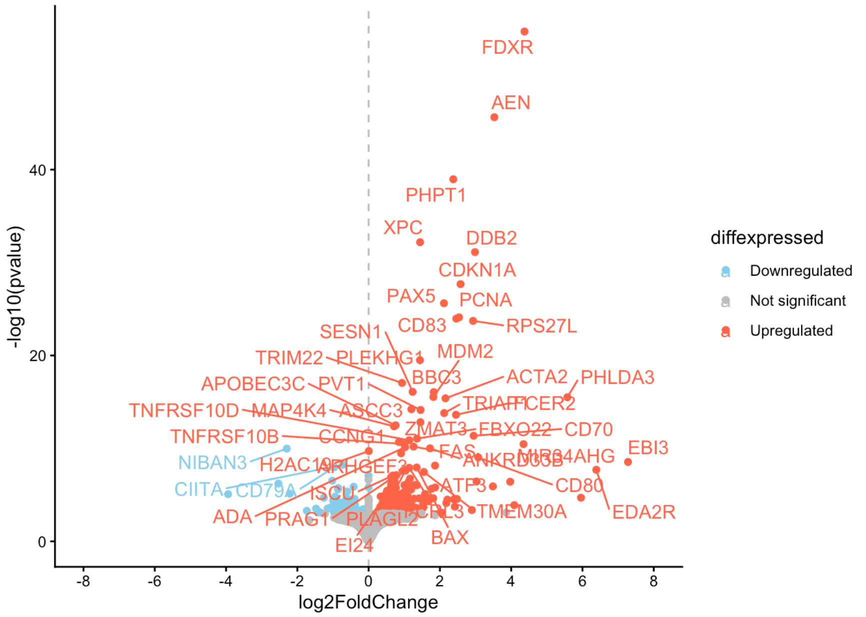

As we can see here, among the highest X-ray sensitive genes upon 2Gy exposure we can find FDXR, CDKN1A, AEN, and CD83.

From this point, we can also perform a functional enrichment analysis to see what pathways are regulated

```
res_enrich_salah <- enrichGO(result_salah_sig$SYMBOL,
                             keyType = "SYMBOL",
                             ont = "BP",
                             OrgDb = "org.Hs.eg.db",
                             pvalueCutoff = 0.05,
                             pAdjustMethod = "BH",
                             qvalueCutoff = 0.2,
                             minGSSize = 10,
                             maxGSSize = 500
)

res_enrich_salah_sig <- res_enrich_salah@result[res_enrich_salah@result$p.adjust <= 0.05, ]
res_enrich_salah_sig |>
  head(20) |>
  knitr::kable()
```

	ID	Description	GeneRatio	BgRatio	RichFactor	FoldEnrichment	zScore	pvalue	p.adjus
GO:0072331	GO:0072331	signal transduction by p53 class mediator	19/239	196/18860	0.0969388	7.649646	10.601833	0	0.0e+0
GO:0097193	GO:0097193	intrinsic apoptotic signaling pathway	24/239	335/18860	0.0716418	5.653407	9.735787	0	0.0e+0
GO:0030098	GO:0030098	lymphocyte differentiation	26/239	441/18860	0.0589569	4.652416	8.792721	0	1.0e-0
GO:0072332	GO:0072332	intrinsic apoptotic signaling pathway by p53 class mediator	13/239	88/18860	0.1477273	11.657474	11.352647	0	1.0e-0

	ID	Description	GeneRatio	BgRatio	RichFactor	FoldEnrichment	zScore	pvalue	p.adjus
GO:0070661	GO:0070661	leukocyte proliferation	23/239	356/18860	0.0646067	5.098256	8.843986	0	1.0e-0
GO:0046651	GO:0046651	lymphocyte proliferation	21/239	312/18860	0.0673077	5.311394	8.699679	0	3.0e-0
GO:0071887	GO:0071887	leukocyte apoptotic process	14/239	125/18860	0.1120000	8.838159	9.960910	0	3.0e-0
GO:0032943	GO:0032943	mononuclear cell proliferation	21/239	319/18860	0.0658307	5.194843	8.560541	0	3.0e-0
GO:0008630	GO:0008630	intrinsic apoptotic signaling pathway in response to DNA damage	13/239	111/18860	0.1171171	9.241962	9.866425	0	6.0e-0
GO:0009411	GO:0009411	response to UV	15/239	159/18860	0.0943396	7.444541	9.245170	0	6.0e-0
GO:0070231	GO:0070231	T cell apoptotic process	10/239	61/18860	0.1639344	12.936415	10.578601	0	1.4e-0
GO:0071214	GO:0071214	cellular response to abiotic stimulus	21/239	355/18860	0.0591549	4.668042	7.904250	0	1.4e-0
GO:0104004	GO:0104004	cellular response to environmental stimulus	21/239	355/18860	0.0591549	4.668042	7.904250	0	1.4e-0
GO:0030330	GO:0030330	DNA damage response, signal transduction by p53 class mediator	11/239	82/18860	0.1341463	10.585774	9.855218	0	1.6e-0
GO:0046631	GO:0046631	alpha-beta T cell activation	16/239	206/18860	0.0776699	6.129098	8.385817	0	1.9e-0
GO:0070227	GO:0070227	lymphocyte apoptotic process	11/239	85/18860	0.1294118	10.212158	9.643566	0	2.1e-0
GO:0034644	GO:0034644	cellular response to UV	11/239	92/18860	0.1195652	9.435146	9.188282	0	4.5e-0
GO:0070663	GO:0070663	regulation of leukocyte proliferation	18/239	284/18860	0.0633803	5.001473	7.697679	0	4.5e-0
GO:0009314	GO:0009314	response to radiation	23/239	462/18860	0.0497835	3.928526	7.220073	0	4.5e-0
GO:0001776	GO:0001776	leukocyte homeostasis	12/239	118/18860	0.1016949	8.024963	8.672291	0	5.7e-0

Now we will also visualize the top 10 regulated pathways.

```
# create GeneTonic object
anno_df <- mosdef::get_annotation_orgdb(
  de_container = dds_salah,
  orgdb_package = "org.Hs.eg.db",
  id_type = "ENSEMBL"
)
#> 'select()' returned 1:many mapping between keys and columns

gtl_salah_6h <- GeneTonic::GeneTonicList(
  dds = dds_salah_6h,
  res_de = results_salah,
  res_enrich = shake_enrichResult(res_enrich_salah),
  annotation_obj = anno_df
)
#> Found 3335 gene sets in `enrichResult` object, of which 276 are significant.
#> Converting for usage in GeneTonic...
#> ---------------------------------
#> ----- GeneTonicList object ------
#> ---------------------------------
#>
#> ----- dds object -----
#> Providing an expression object (as DESeqDataset) of 62646 features over 15 samples
```

```
#>
#> ----- res_de object -----
#> Providing a DE result object (as DESeqResults), 62646 features tested, 274 found as DE
#> Upregulated:     197
#> Downregulated:   77
#>
#> ----- res_enrich object -----
#> Providing an enrichment result object, 3335 reported
#>
#> ----- annotation_obj object -----
#> Providing an annotation object of 62646 features with information on 2 identifier types

# preparing the matrix for heatmap
scored_mat <- GeneTonic::gs_scores(se = vsd_corrected, gtl = gtl_salah_6h)

anno <- as.data.frame(colData(vsd_corrected)[, c("Sex", "Dose")])
dose_colors <- colorRampPalette(c("lightyellow", "gold", "orange", "red"))(5)
names(dose_colors)<- c("0Gy", "0.5Gy", "1Gy", "2Gy", "4Gy")
anno_colors <- list(
  Sex = c(Female = "pink", Male = "blue"),
  Dose = dose_colors
)
column_annotation <- HeatmapAnnotation(
  Sex = anno$Sex,
  Dose = anno$Dose,
  col = anno_colors, show_legend = TRUE
)

col_fun <- colorRamp2(
  c(-2, 0, 2),
  c("blue", "white", "red")
)

ht <- ComplexHeatmap::Heatmap(scored_mat[1:20,],
  name = "Expression",
  top_annotation = column_annotation,
  cluster_columns = FALSE, # Optional: Cluster columns
  cluster_rows = FALSE, # Optional: Cluster rows
  show_row_names = TRUE,
  show_column_names = TRUE,
  row_dend_side = "left",
  row_names_side = "left",
  row_names_gp = gpar(fontsize = 5, fontface= "bold" ),
  width = unit(7, "cm"),
  heatmap_height = unit(12, "cm"),
  column_names_gp = gpar(fontsize = 7),
  column_names_rot = 90,
  column_title = "Donors",
  column_title_side = "bottom",
  show_heatmap_legend = TRUE,
  col = col_fun
)

draw(ht, heatmap_legend_side = "right", annotation_legend_side = "right")
```

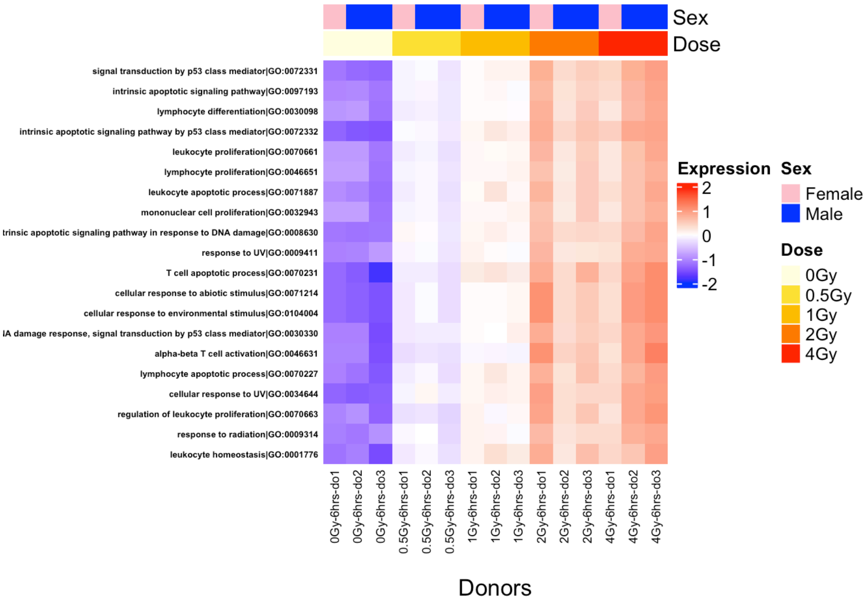

TODO; We can see here that among the top 20 regulated pathways we can find processes such as DNA damage repair, immune response, cellular response to UV, and some other associated GO terms.

9.2 Working on the gamma ray dataset (Paul et al. 2013)

We might also want to compare Xray signature to gamma ray signatures, to see if both ionizing radiation types have shared and distinct transcriptional responses we will therefore focus on the `se_paul` dataset, since it has the same time point of `se_salah` (6 hours after irradiation).

In this `se_paul` dataset, Age and Sex should be accounted for to capture signals only in response to radiation

```
# for comparability, we will only focus on 6hr timepoint
se_paul_6h <- se_paul[, colData(se_paul)$Time_point == "6hr"]
# since it is a repeated measurements experiment, we will create a pseudo-donor factor to acco

expr_values <- assay(se_paul_6h)
# remove probes with NA values
expr_values <- expr_values[rowSums(is.na(expr_values)) == 0, ]

# remove probes with zero variance
expr_values <- expr_values[apply(expr_values, 1, sd) != 0, ]

# extracting metadata
meta_df <- as.data.frame(colData(se_paul_6h))

# number of donors
n_donors <- 5
# number of doses per donor
n_doses <- 5

# create pseudo-donor factor
meta_df$Donors <- factor(rep(1:n_donors, each = n_doses))

# check
table(meta_df$Donors)
#>
#> 1 2 3 4 5
#> 5 5 5 5 5

# handle the covariates as factors
meta_df$Dose <- factor(meta_df$Dose)
meta_df$Dose <- relevel(meta_df$Dose, ref = "0Gy")
meta_df$Sex  <- factor(meta_df$Sex)
meta_df$Age <- as.numeric(gsub("[^0-9]", "", meta_df$Age))

# building the design matrix
design_paul <- model.matrix(~ Age + Sex + Dose, data = meta_df)
design_paul
#>            (Intercept) Age Sexmale Dose0.5Gy Dose2Gy Dose5Gy Dose8Gy
#> GSM1080589           1  39       1         1       0       0       0
#> GSM1080590           1  39       1         0       0       0       0
#> GSM1080591           1  39       1         0       1       0       0
#> GSM1080592           1  39       1         0       0       1       0
#> GSM1080593           1  39       1         0       0       0       1
#> GSM1080594           1  20       0         1       0       0       0
#> GSM1080595           1  20       0         0       0       0       0
#> GSM1080596           1  20       0         0       1       0       0
#> GSM1080597           1  20       0         0       0       1       0
#> GSM1080598           1  20       0         0       0       0       1
#> GSM1080599           1  53       1         1       0       0       0
#> GSM1080600           1  53       1         0       0       0       0
#> GSM1080601           1  53       1         0       1       0       0
#> GSM1080602           1  53       1         0       0       1       0
#> GSM1080603           1  53       1         0       0       0       1
#> GSM1080604           1  35       0         1       0       0       0
#> GSM1080605           1  35       0         0       0       0       0
#> GSM1080606           1  35       0         0       1       0       0
#> GSM1080607           1  35       0         0       0       1       0
#> GSM1080608           1  35       0         0       0       0       1
#> GSM1080609           1  36       0         1       0       0       0
#> GSM1080610           1  36       0         0       0       0       0
#> GSM1080611           1  36       0         0       1       0       0
#> GSM1080612           1  36       0         0       0       1       0
#> GSM1080613           1  36       0         0       0       0       1
#> attr(,"assign")
#> [1] 0 1 2 3 3 3 3
#> attr(,"contrasts")
#> attr(,"contrasts")$Sex
#> [1] "contr.treatment"
#>
#> attr(,"contrasts")$Dose
#> [1] "contr.treatment"

# blocking for the donor effect
corfit_paul <- duplicateCorrelation(expr_values, design_paul, block = meta_df$Donors)
```

```r
# fitting the model
fit_paul <- lmFit(expr_values, design_paul, block = meta_df$Patient, correlation = corfit_paul

contrast.matrix_paul <- makeContrasts(Dose2Gy, levels = design_paul)
#> Renaming (Intercept) to Intercept
fit2_paul <- contrasts.fit(fit_paul, contrast.matrix_paul)
fit2_paul <- eBayes(fit2_paul)

# extracting the results
results_paul <- topTable(fit2_paul, number = Inf)
results_paul_sig <- results_paul[ results_paul$adj.P.Val<= 0.05,]

results_paul_sig |>
  head(10) |>
  knitr::kable()
```

	logFC	AveExpr	t	P.Value	adj.P.Val	B
FDXR	5.114015	11.828418	45.28673	0	0	31.89617
DDB2	2.971409	11.710745	28.29392	0	0	27.42778
CD70	3.501244	9.363679	23.68030	0	0	25.17089
PCNA	2.691635	9.432219	21.75109	0	0	23.99659
_A_24_P127462	2.900812	8.107424	21.66597	0	0	23.94103
RPS27L	1.994590	11.034623	18.90364	0	0	21.94062
GADD45A	2.100923	9.198538	17.26089	0	0	20.54319
_A_32_P85230	3.472482	8.768788	17.00659	0	0	20.31096
SESN1_A_23_P93562	1.806004	8.151917	16.02157	0	0	19.36746
DCP1B	1.271772	9.040659	15.50149	0	0	18.83951

Here, we will extract the gene symbol part without the probe id so we can compare the genes with the Salah_2025 dataset

```r
results_paul_sig$SYMBOL <- sub("_.*", "", rownames(results_paul_sig))

# again, we can also check this effect at the pathway level
res_enrich_paul <- enrichGO(results_paul_sig$SYMBOL,
                            keyType = "SYMBOL",
                            ont = "BP",
                            OrgDb = "org.Hs.eg.db",
                            pvalueCutoff = 0.05,
                            pAdjustMethod = "BH",
                            qvalueCutoff = 0.2,
                            minGSSize = 10,
                            maxGSSize = 500
)

res_enrich_paul_sig <- res_enrich_paul@result[res_enrich_paul@result$p.adjust <= 0.05, ]

res_enrich_paul_sig |>
  head(20) |>
  knitr::kable()
```

	ID	Description	GeneRatio	BgRatio	RichFactor	FoldEnrichment	zScore	pvalue	p.adju
GO:0097193	GO:0097193	intrinsic apoptotic signaling pathway	18/95	335/18860	0.0537313	10.667086	12.702398	0	0e+0
GO:0071214	GO:0071214	cellular response to abiotic stimulus	18/95	355/18860	0.0507042	10.066123	12.269817	0	0e+0
GO:0104004	GO:0104004	cellular response to environmental stimulus	18/95	355/18860	0.0507042	10.066123	12.269817	0	0e+0
GO:0034644	GO:0034644	cellular response to UV	11/95	92/18860	0.1195652	23.736842	15.554710	0	0e+0
GO:0009411	GO:0009411	response to UV	13/95	159/18860	0.0817610	16.231711	13.723399	0	0e+0
GO:0071478	GO:0071478	cellular response to radiation	13/95	188/18860	0.0691489	13.727884	12.479194	0	0e+0

	ID	Description	GeneRatio	BgRatio	RichFactor	FoldEnrichment	zScore	pvalue	p.adju
GO:0009314	GO:0009314	response to radiation	18/95	462/18860	0.0389610	7.734792	10.428136	0	0e+0
GO:2001242	GO:2001242	regulation of intrinsic apoptotic signaling pathway	13/95	195/18860	0.0666667	13.235088	12.219607	0	0e+0
GO:0072331	GO:0072331	signal transduction by p53 class mediator	13/95	196/18860	0.0663265	13.167562	12.183613	0	0e+0
GO:0072332	GO:0072332	intrinsic apoptotic signaling pathway by p53 class mediator	10/95	88/18860	0.1136364	22.559809	14.423738	0	0e+0
GO:0071482	GO:0071482	cellular response to light stimulus	11/95	124/18860	0.0887097	17.611205	13.204453	0	0e+0
GO:0008630	GO:0008630	intrinsic apoptotic signaling pathway in response to DNA damage	10/95	111/18860	0.0900901	17.885254	12.694830	0	0e+0
GO:0030330	GO:0030330	DNA damage response, signal transduction by p53 class mediator	9/95	82/18860	0.1097561	21.789474	13.423711	0	1e-0
GO:0070227	GO:0070227	lymphocyte apoptotic process	9/95	85/18860	0.1058824	21.020433	13.162543	0	1e-0
GO:0071887	GO:0071887	leukocyte apoptotic process	10/95	125/18860	0.0800000	15.882105	11.877892	0	1e-0
GO:0070231	GO:0070231	T cell apoptotic process	8/95	61/18860	0.1311475	26.036238	13.935208	0	1e-0
GO:0070228	GO:0070228	regulation of lymphocyte apoptotic process	8/95	63/18860	0.1269841	25.209691	13.695002	0	1e-0
GO:2000106	GO:2000106	regulation of leukocyte apoptotic process	9/95	97/18860	0.0927835	18.419967	12.238520	0	2e-0
GO:0009416	GO:0009416	response to light stimulus	14/95	341/18860	0.0410557	8.150640	9.481133	0	2e-0
GO:2001233	GO:2001233	regulation of apoptotic signaling pathway	15/95	406/18860	0.0369458	7.334716	9.181038	0	2e-0

Similarly to X-ray dataset from `se_salah`, many of the regulated pathways were involved in processes such as DNA damage repair, immune response, and response to UV.

10 Integrating the results from both studies

Checking the shared genes between both radiation types, at the same timepoint and radiation dose

```
result_salah_sig<- na.omit(result_salah_sig)

# creating a simple venn diagram first
venn.plot <- venn.diagram(
  x = list(
    Salah = result_salah_sig$SYMBOL,
    Paul = results_paul_sig$SYMBOL
  ),
  fill = c("#8da0cb", "orange"),
  alpha = 0.6,
```

```r
  cat.cex = 1.2,
  cex = 1.5,
  main = "Overlap of DE genes - Shared and distinct signatures",
  filename = NULL,
  disable.logging = TRUE)

grid::grid.draw(venn.plot)
```

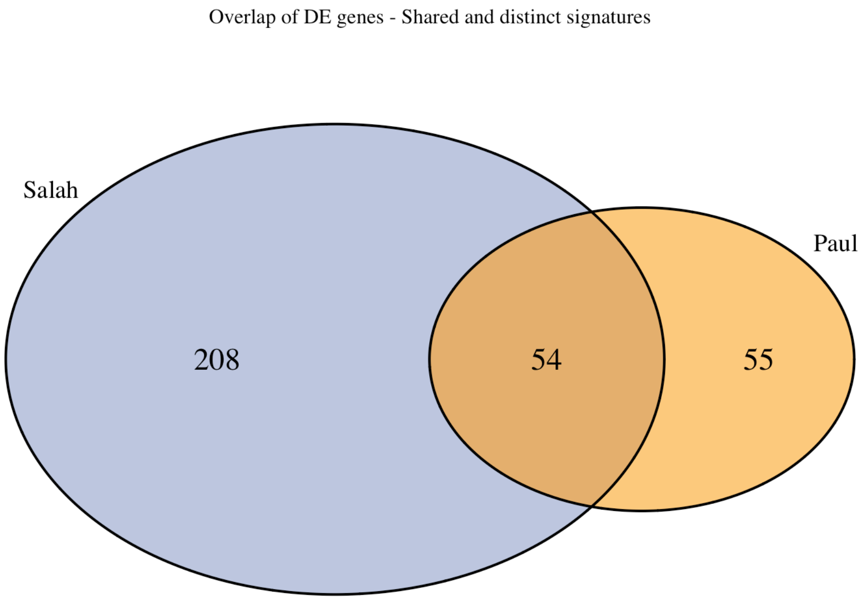


```r
# shared signature between X and gamma rays
intersect(result_salah_sig$SYMBOL, results_paul_sig$SYMBOL)
#>  [1] "REV3L"    "FAS"      "PHPT1"    "BCL3"     "NFKB2"    "SESN1"
#>  [7] "BAX"      "IZUMO4"   "CD40"     "RELB"     "EBI3"     "BBC3"
#> [13] "CD79A"    "ACTA2"    "SYNGR2"   "CD83"     "PTP4A1"   "ASCC3"
#> [19] "TMEM30A"  "CCNG1"    "GADD45A"  "TNFRSF10B" "CDKN1A"   "CD70"
#> [25] "PLAGL2"   "CCR7"     "GDF15"    "TRIM22"   "PCNA"     "DDB2"
#> [31] "MDM2"     "DRAM1"    "MYC"      "PMAIP1"   "FGD2"     "EI24"
#> [37] "DCP1B"    "BCL2L11"  "XPC"      "FDXR"     "IER5"     "ARHGEF3"
#> [43] "FBXO22"   "VWCE"     "POLH"     "PPM1D"    "TRIAP1"   "BCL2"
#> [49] "ZMAT3"    "RPS27L"   "ADA"      "ANXA4"    "APOBEC3C" "MARCKS"

# gamma-ray only signature
setdiff(results_paul_sig$SYMBOL, result_salah_sig$SYMBOL)
#>  [1] ""         "FAM129C"  "ANKRA2"   "PLK3"     "C12orf5"  "CCDC90B"
#>  [7] "MGAT3"    "LIG1"     "C17orf89" "ZNF337"   "METTL7A"  "PLK2"
#> [13] "IL21R"    "SERPINB1" "TTC21A"   "GPR84"    "TNIP1"    "LY9"
#> [19] "C3orf26"  "SLC7A6"   "APOBEC3F" "THOP1"    "RGS12"    "SCRIB"
#> [25] "PTGS2"    "GHRL"     "PYCR1"    "TNFAIP6"  "P2RX5"    "ZMIZ2"
#> [31] "PLEKHF1"  "SAC3D1"   "FAM136A"  "SGK223"   "UPB1"     "METTL12"
#> [37] "RFTN1"    "IL8"      "TNFRSF12A" "ID2"     "BTG3"     "JAK3"
#> [43] "MR1"      "ZNF195"   "MRPL49"   "TMEM205"  "TM7SF3"   "TESK2"
#> [49] "SNHG12"   "FBXW7"    "NAP1L1"   "TP53TG1"  "POPDC2"   "TSPAN32"
#> [55] "ERN1"

# X-ray only signature
setdiff(result_salah_sig$SYMBOL, results_paul_sig$SYMBOL)
#>   [1] "HIVEP2"     "ALOX5"      "TACC3"      "BIRC3"      "DEF6"
#>   [6] "UBA6"       "DAPK2"      "PRDM1"      "RASGRF1"    "TBXAS1"
#>  [11] "CALCRL"     "IDI1"       "MAP4K4"     "ACTN1"      "ACAP1"
#>  [16] "ZNF37A"     "PLD1"       "TRAF4"      "TIGAR"      "TP53INP2"
#>  [21] "EPB41L2"    "PCNP"       "COBLL1"     "PIBF1"      "CYLD"
#>  [26] "ATRN"       "SLC8B1"     "HIVEP1"     "PPIL2"      "APOBEC3H"
#>  [31] "CSF2RB"     "HDAC10"     "HIF1A"      "PPP4R3A"    "CCM2L"
#>  [36] "TM9SF4"     "RBBP8"      "RENBP"      "JADE3"      "ACP5"
#>  [41] "N4BP1"      "CCL22"      "GGA2"       "NBN"        "FCER2"
#>  [46] "ASF1B"      "DBP"        "GSK3A"      "SNX8"       "TNFSF8"
#>  [51] "LIPA"       "GBF1"       "UNC119"     "NFKB1"      "DBN1"
#>  [56] "GORASP1"    "IFIH1"      "RALGPS2"    "RNF19B"     "WLS"
#>  [61] "SIPA1L2"    "TNFSF4"     "PLAGL1"     "TNFAIP3"    "SGK1"
#>  [66] "ATL2"       "PANK3"      "PLEKHG1"    "CD80"       "ZC3H7A"
#>  [71] "MRM2"       "KCNJ2"      "PI3"        "PLCG1"      "MIF4GD"
#>  [76] "TNFSF9"     "MED1"       "CD93"       "IQCN"       "PNPLA7"
#>  [81] "HIP1R"      "EDA2R"      "TRAF3"      "NPHP4"      "PTGFRN"
#>  [86] "NOTCH2"     "IL2RA"      "GLS2"       "NHSL1"      "ISCU"
#>  [91] "LIMD2"      "IRF4"       "MMP7"       "ACVRL1"     "NKD1"
#>  [96] "FN3KRP"     "IGFBP4"     "SLC2A5"     "RGL1"       "GOLGA4"
```

```
#> [101] "LPP"          "TIFA"         "FEZ1"         "MERTK"        "NDUFAF6"
#> [106] "ZDHHC5"       "ZFAND3"       "MS4A1"        "ODR4"         "SNX22"
#> [111] "PANK4"        "RNF207"       "TSPAN33"      "B4GALT5"      "CD1D"
#> [116] "CD1C"         "CCAR2"        "SON"          "TNFRSF13C"    "PTGIR"
#> [121] "JAML"         "PCSK7"        "FCRL3"        "PWWP3A"       "ABCF3"
#> [126] "LRP5"         "MEGF6"        "PEA15"        "ATF3"         "REL"
#> [131] "SMC6"         "CDC42EP3"     "PYHIN1"       "UVSSA"        "SLBP"
#> [136] "ANKRD33B"     "CEP295"       "ABTB2"        "TCP11L2"      "NOLC1"
#> [141] "ZCCHC18"      "DPEP2"        "NIBAN3"       "TAPT1"        "C11orf24"
#> [146] "LRG1"         "WIPF2"        "PTGER4"       "PIK3CD"       "GPR82"
#> [151] "SYNPO"        "FRMD3"        "CEBPB"        "TRIB1"        "TNFRSF10D"
#> [156] "EIF1AX"       "PHLDA3"       "CKAP5"        "P2RY2"        "MLXIP"
#> [161] "GNG7"         "ACER2"        "PTPN11"       "CIITA"        "LACC1"
#> [166] "AEN"          "ZNF746"       "FRAT2"        "TSEN54"       "CCDC125"
#> [171] "TNFAIP2"      "L3MBTL1"      "IER5L"        "FAM111B"      "PAX5"
#> [176] "ZNF79"        "ZNF431"       "KIAA1671"     "SDAD1"        "BAZ1A"
#> [181] "MDM4"         "RUSC2"        "BRD2"         "IGKC"         "CEBPD"
#> [186] "LOC102723566" "FAM30A"       "MIR34AHG"     "TRAF3IP2-AS1" "MIR155HG"
#> [191] "TMEM250"      "IGKV1-33"     "NAIP"         "PVT1"         "IGKV1D-39"
#> [196] "CORO7"        "CCL4"         "PRAG1"        "CCL4L2"       "GTSE1-DT"
#> [201] "CCL3"         "ZNF229"       "LOC124901671" "SMIM10L3"     "H2AC19"
#> [206] "LOC100294145" "NSUN5P1"      "GTF2IP4"
```

Another way to display the shared and distinct regulation happening at the overall transcriptome level would be a logFC-logFC plot - of course, with the caveat of these two datasets originating from two different technology platforms (RNA-seq for `se_salah` and microarray for `se_paul`)

```
dim(results_salah_shrink)
#> [1] 62646     5
dim(results_paul)
#> [1] 14936     6

results_salah_shrink$SYMBOL <- mapIds(
  org.Hs.eg.db,
  keys = rownames(results_salah_shrink),
  column = "SYMBOL",
  keytype = "ENSEMBL",
  multiVals = "first"
)
#> 'select()' returned 1:many mapping between keys and columns

results_paul$SYMBOL <- rownames(results_paul)

merged_results <- merge.data.frame(x = as.data.frame(results_salah_shrink),
                                   y = results_paul,
                                   by = "SYMBOL")
merged_results$DE_salah <- merged_results$padj <= 0.05 & !is.na(merged_results$padj)
merged_results$DE_paul <- merged_results$adj.P.Val <= 0.05

ggplot(merged_results,
       aes(x = log2FoldChange, y = logFC, col = DE_salah)) +
  geom_point() +
  scale_color_manual(values = c(scales::alpha("black", 0.3), "firebrick")) +
  theme_bw() +
  labs(x = "logFC - RNA-seq",
       y = "logFC - microarray",
       title = "Highlighting DE genes from Salah et al.")
#> Warning: Removed 123 rows containing missing values or values outside the scale range
#> (`geom_point()`).
```

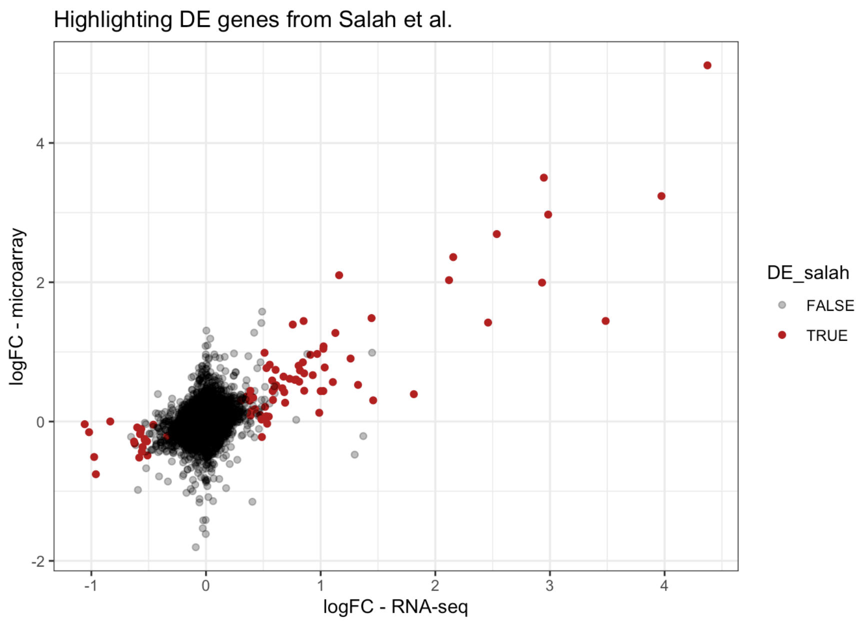


```
ggplot(merged_results,
         aes(x = log2FoldChange, y = logFC, col = DE_paul)) +
  geom_point() +
  scale_color_manual(values = c(scales::alpha("black", 0.3), "firebrick")) +
  theme_bw() +
  labs(x = "logFC − RNA−seq",
       y = "logFC − microarray",
       title = "Highlighting DE genes from Paul et al.")
#> Warning: Removed 123 rows containing missing values or values outside the scale range
#> (`geom_point()`).
```

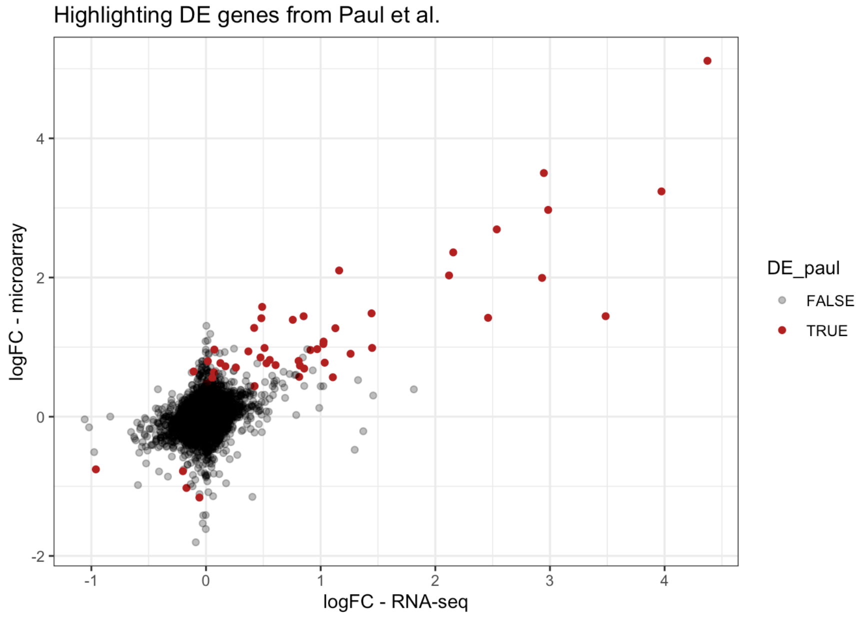

We can appreciate better how the gene expression regulation is to be assessed in quantitative nuances that overcome the dichotomization between significance or not, and the scatter plot of effect sizes can also better highlight the concordance in regulation direction.

One can also easily plot a geneset signature derived from one dataset (e.g. GO:0009411, response to UV, found in the microarray analysis), using the expression values of the other (Salah et al.).

```
genes_uvresponse_paul <- res_enrich_paul_sig[res_enrich_paul_sig$ID == "GO:0009411", "geneID"]
  strsplit(split = "/") |>
  unlist()

genes_uvresponse_paul_ensembl <- mapIds(org.Hs.eg.db,
                                        keys = genes_uvresponse_paul,
                                        column = "ENSEMBL",
                                        keytype = "SYMBOL")
#> 'select()' returned 1:1 mapping between keys and columns

GeneTonic::gs_heatmap(se = vsd_corrected,
```

```
                      gtl = gtl_salah_6h,
                      genelist = genes_uvresponse_paul_ensembl,
                      plot_title = "Using the DE genes from Paul et al.")
```

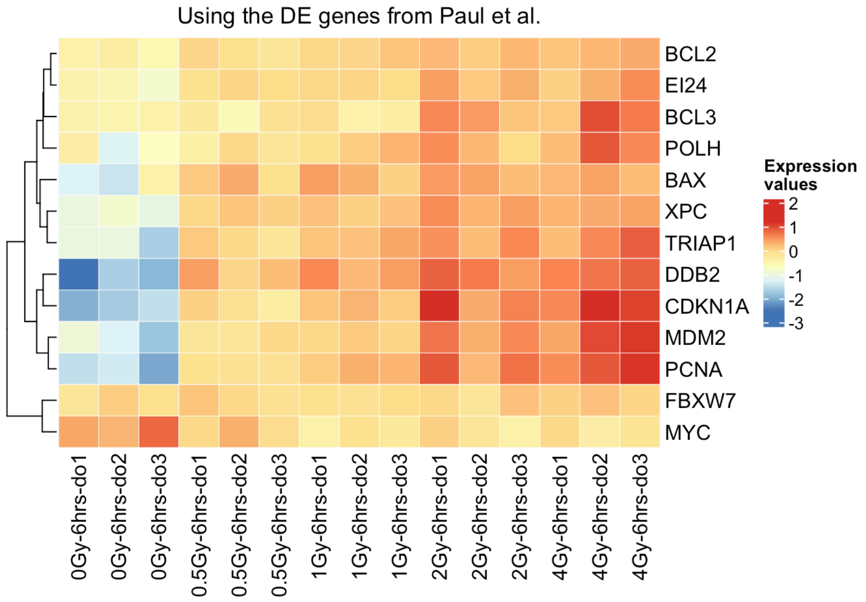


```
GeneTonic::gs_heatmap(se = vsd_corrected,
                      gtl = gtl_salah_6h,
                      geneset_id = "GO:0009411",
                      plot_title = "Using the original DE genes for UV response from Salah et
```

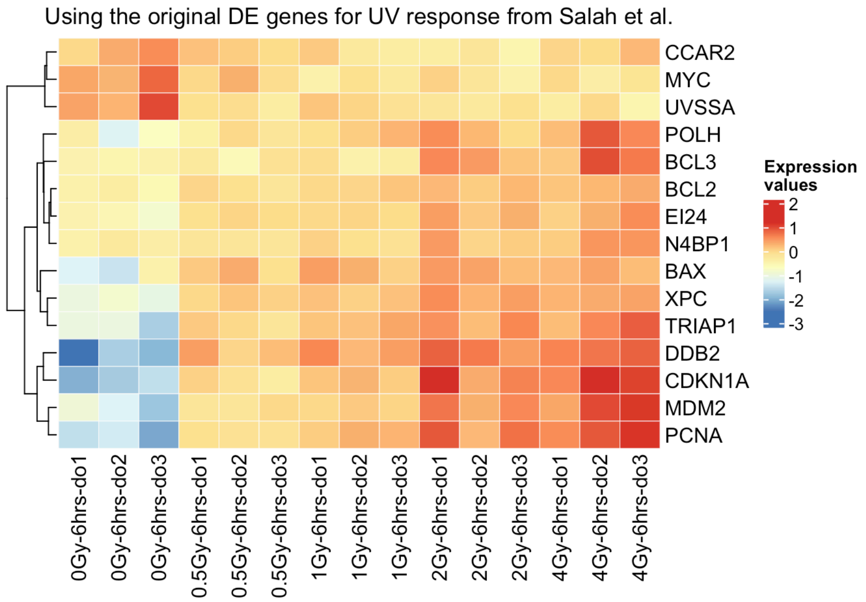

While most of the genes are shared, the higher power in DE detection from Salah et al. made it possible to identify a more comprehensive signature linked to this biological process.

Similarly to what we have just showcased, it is possible to compare other genesets and signatures across the individual datasets, e.g. apoptosis, response to DNA damage, and immune responses.

11 Wrapping up

This notebook can be further expanded to include additional datasets - e.g., scenarios where some discordant regulation is expected. Having `DoReMiTra` providing a number of them in this analysis-ready format makes it easy to explore them in detail, ultimately better understanding the transcriptional changes occurring upon radiation.

Overall, data integration and comparison through well-curated radiation transcriptomic datasets available through the `DoReMiTra` collection is useful in biomarker discovery upon radiation exposure.

Session info

```
sessionInfo()
#> R Under development (unstable) (2026-02-10 r89394)
#> Platform: x86_64-apple-darwin20
#> Running under: macOS Sequoia 15.7.3
#> 
#> Matrix products: default
#> BLAS:   /Library/Frameworks/R.framework/Versions/4.6-x86_64/Resources/lib/libRblas.0.dylib
#> LAPACK: /Library/Frameworks/R.framework/Versions/4.6-x86_64/Resources/lib/libRlapack.dylib;
#> 
#> locale:
#> [1] en_US.UTF-8/en_US.UTF-8/en_US.UTF-8/C/en_US.UTF-8/en_US.UTF-8
#> 
#> time zone: Europe/Berlin
#> tzcode source: internal
#> 
#> attached base packages:
#> [1] grid      stats4    stats     graphics  grDevices utils     datasets 
#> [8] methods   base     
#> 
#> other attached packages:
#>  [1] DoReMiTraExplorer_1.2.0     patchwork_1.3.2            
#>  [3] clusterProfiler_4.19.1      GeneTonic_3.5.0            
#>  [5] circlize_0.4.17             limma_3.67.0               
#>  [7] VennDiagram_1.8.2           futile.logger_1.4.9        
#>  [9] ComplexHeatmap_2.27.1       ggplot2_4.0.2              
#> [11] DESeq2_1.51.6               SummarizedExperiment_1.41.1
#> [13] MatrixGenerics_1.23.0       matrixStats_1.5.0          
#> [15] GenomicRanges_1.63.1        Seqinfo_1.1.0              
#> [17] org.Hs.eg.db_3.22.0         AnnotationDbi_1.73.0       
#> [19] IRanges_2.45.0              S4Vectors_0.49.0           
#> [21] Biobase_2.71.0              BiocGenerics_0.57.0        
#> [23] generics_0.1.4              mosdef_1.7.0               
#> [25] DoReMiTra_1.1.2            
#> 
#> loaded via a namespace (and not attached):
#>   [1] fs_1.6.6                    bitops_1.0-9               
#>   [3] enrichplot_1.31.0           httr_1.4.7                 
#>   [5] RColorBrewer_1.1-3          doParallel_1.0.17          
#>   [7] numDeriv_2016.8-1.1         dynamicTreeCut_1.63-1      
#>   [9] tippy_0.1.0                 tools_4.6.0                
#>  [11] R6_2.6.1                    DT_0.34.0                  
#>  [13] lazyeval_0.2.2              mgcv_1.9-4                 
#>  [15] apeglm_1.33.0               GetoptLong_1.1.0           
#>  [17] withr_3.0.2                 prettyunits_1.2.0          
#>  [19] gridExtra_2.3               textshaping_1.0.4          
#>  [21] cli_3.6.5                   formatR_1.14               
#>  [23] Cairo_1.7-0                 labeling_0.4.3             
#>  [25] sass_0.4.10                 topGO_2.63.0               
#>  [27] bs4Dash_2.3.5               mvtnorm_1.3-3              
#>  [29] S7_0.2.1                    ggridges_0.5.7             
#>  [31] goseq_1.63.0                Rsamtools_2.27.0           
#>  [33] systemfonts_1.3.1           yulab.utils_0.2.4          
#>  [35] gson_0.1.0                  txdbmaker_1.7.3            
#>  [37] svglite_2.2.2               DOSE_4.5.0                 
#>  [39] scater_1.39.2               dichromat_2.0-0.1          
#>  [41] bbmle_1.0.25.1              rstudioapi_0.18.0          
#>  [43] RSQLite_2.4.6               visNetwork_2.1.4           
#>  [45] gridGraphics_0.5-1          shape_1.4.6.1              
#>  [47] BiocIO_1.21.0               dplyr_1.2.0                
#>  [49] dendextend_1.19.1           GO.db_3.22.0               
#>  [51] Matrix_1.7-4                ggbeeswarm_0.7.3           
#>  [53] abind_1.4-8                 lifecycle_1.0.5            
#>  [55] yaml_2.3.12                 qvalue_2.43.0              
#>  [57] SparseArray_1.11.10         BiocFileCache_3.1.0        
#>  [59] blob_1.3.0                  promises_1.5.0             
#>  [61] bdsmatrix_1.3-7             ExperimentHub_3.1.0        
#>  [63] crayon_1.5.3                miniUI_0.1.2               
#>  [65] ggtangle_0.1.1              lattice_0.22-9             
#>  [67] beachmat_2.27.2             ComplexUpset_1.3.6         
#>  [69] cowplot_1.2.0               GenomicFeatures_1.63.1     
#>  [71] cigarillo_1.1.0             KEGGREST_1.51.1            
#>  [73] magick_2.9.0                pillar_1.11.1              
#>  [75] knitr_1.51                  fgsea_1.37.4               
#>  [77] rjson_0.2.23                codetools_0.2-20           
#>  [79] fastmatch_1.1-8             glue_1.8.0                 
#>  [81] ggiraph_0.9.4               ggfun_0.2.0                
#>  [83] fontLiberation_0.1.0        data.table_1.18.2.1        
#>  [85] vctrs_0.7.1                 png_0.1-8                  
#>  [87] treeio_1.35.0               gtable_0.3.6               
```

```
#>  [89] emdbook_1.3.14              cachem_1.1.0
#>  [91] xfun_0.56                   S4Arrays_1.11.1
#>  [93] mime_0.13                   coda_0.19-4.1
#>  [95] SingleCellExperiment_1.33.0 iterators_1.0.14
#>  [97] statmod_1.5.1               nlme_3.1-168
#>  [99] ggtree_4.1.1                bit64_4.6.0-1
#> [101] fontquiver_0.2.1            progress_1.2.3
#> [103] filelock_1.0.3              GenomeInfoDb_1.47.2
#> [105] bslib_0.10.0                irlba_2.3.7
#> [107] vipor_0.4.7                 otel_0.2.0
#> [109] colorspace_2.1-2            DBI_1.2.3
#> [111] tidyselect_1.2.1            bit_4.6.0
#> [113] compiler_4.6.0              curl_7.0.0
#> [115] httr2_1.2.2                 graph_1.89.1
#> [117] BiocNeighbors_2.5.3         BiasedUrn_2.0.12
#> [119] SparseM_1.84-2              expm_1.0-0
#> [121] xml2_1.5.2                  fontBitstreamVera_0.1.1
#> [123] DelayedArray_0.37.0         plotly_4.12.0
#> [125] colourpicker_1.3.0          rtracklayer_1.71.3
#> [127] scales_1.4.0                rappdirs_0.3.4
#> [129] stringr_1.6.0               digest_0.6.39
#> [131] rmarkdown_2.30              XVector_0.51.0
#> [133] htmltools_0.5.9             pkgconfig_2.0.3
#> [135] dbplyr_2.5.1                fastmap_1.2.0
#> [137] rlang_1.1.7                 GlobalOptions_0.1.3
#> [139] htmlwidgets_1.6.4           UCSC.utils_1.7.1
#> [141] shiny_1.12.1                jquerylib_0.1.4
#> [143] farver_2.1.2                jsonlite_2.0.0
#> [145] BiocParallel_1.45.0         GOSemSim_2.37.2
#> [147] BiocSingular_1.27.1         RCurl_1.98-1.17
#> [149] magrittr_2.0.4              kableExtra_1.4.0
#> [151] scuttle_1.21.0              ggplotify_0.1.3
#> [153] Rcpp_1.1.1                  shinycssloaders_1.1.0
#> [155] ape_5.8-1                   viridis_0.6.5
#> [157] gdtools_0.5.0               rintrojs_0.3.4
#> [159] stringi_1.8.7               MASS_7.3-65
#> [161] AnnotationHub_4.1.0         plyr_1.8.9
#> [163] parallel_4.6.0              ggrepel_0.9.6
#> [165] Biostrings_2.79.4           splines_4.6.0
#> [167] hms_1.1.4                   geneLenDataBase_1.47.0
#> [169] locfit_1.5-9.12             igraph_2.2.1
#> [171] ScaledMatrix_1.19.0         reshape2_1.4.5
#> [173] biomaRt_2.67.1              futile.options_1.0.1
#> [175] BiocVersion_3.23.1          XML_3.99-0.22
#> [177] evaluate_1.0.5              lambda.r_1.2.4
#> [179] BiocManager_1.30.27         foreach_1.5.2
#> [181] tweenr_2.0.3                httpuv_1.6.16
#> [183] backbone_3.0.3              tidyr_1.3.2
#> [185] purrr_1.2.1                 polyclip_1.10-7
#> [187] clue_0.3-66                 ggforce_0.5.0
#> [189] rsvd_1.0.5                  xtable_1.8-4
#> [191] restfulr_0.0.16             tidytree_0.4.7
#> [193] later_1.4.5                 viridisLite_0.4.3
#> [195] tibble_3.3.1                aplot_0.2.9
#> [197] beeswarm_0.4.0              memoise_2.0.1
#> [199] GenomicAlignments_1.47.0    cluster_2.1.8.2
#> [201] shinyWidgets_0.9.0          shinyAce_0.4.4
```

References

Paul, Sunirmal, Lubomir B. Smilenov, and Sally A. Amundson. 2013. “Widespread Decreased Expression of Immune Function Genes in Human Peripheral Blood Following Radiation Exposure.” *Radiation Research* 180 (6): 575. https://doi.org/10.1667/rr13343.1.

Salah, Ahmed, Daniel Wollschläger, Maurizio Callari, Heinz Schmidberger, Federico Marini, and Sebastian Zahnreich. 2025. “Genome-Wide Transcriptomic Response of Whole Blood to Radiation.” *Scientific Reports* 15 (1). https://doi.org/10.1038/s41598-025-04898-1.
